# Elucidating Self‐Assembling Peptide Aggregation via Morphoscanner: A New Tool for Protein‐Peptide Structural Characterization

**DOI:** 10.1002/advs.201800471

**Published:** 2018-06-22

**Authors:** Gloria A. A. Saracino, Federico Fontana, Shehrazade Jekhmane, João Medeiros Silva, Markus Weingarth, Fabrizio Gelain

**Affiliations:** ^1^ Center for Nanomedicine and Tissue Engineering (CNTE) ASST Ospedale Niguarda Cà Granda Piazza dell'Ospedale Maggiore 3 20162 Milan Italy; ^2^ IRCCS Casa Sollievo della Sofferenza Opera di San Pio da Pietralcina Viale Capuccini 1 71013 San Giovanni Rotondo Italy; ^3^ NMR Spectroscopy Bijvoet Center for Biomolecular Research Department of Chemistry Utrecht University Padualaan 8 3584 CH Utrecht The Netherlands

**Keywords:** β‐structures, coarse‐grained molecular dynamics, multilayer graph theory, pattern recognition, self‐assembling peptides

## Abstract

Self‐assembling and molecular folding are ubiquitous in Nature: they drive the organization of systems ranging from living creatures to DNA molecules. Elucidating the complex dynamics underlying these phenomena is of crucial importance. However, a tool for the analysis of the various phenomena involved in protein/peptide aggregation is still missing. Here, an innovative software is developed and validated for the identification and visualization of *b*‐structuring and *b*‐sheet formation in both simulated systems and crystal structures of proteins and peptides. The novel software suite, dubbed Morphoscanner, is designed to identify and intuitively represent *b*‐structuring and *b*‐sheet formation during molecular dynamics trajectories, paying attention to temporary strand‐strand alignment, suboligomer formation and evolution of local order. Self‐assembling peptides (SAPs) constitute a promising class of biomaterials and an interesting model to study the spontaneous assembly of molecular systems in vitro. With the help of coarse‐grained molecular dynamics the self‐assembling of diverse SAPs is simulated into molten aggregates. When applied to these systems, Morphoscanner highlights different *b*‐structuring schemes and kinetics related to SAP sequences. It is demonstrated that Morphoscanner is a novel versatile tool designed to probe the aggregation dynamics of self‐assembling systems, adaptable to the analysis of differently coarsened simulations of a variety of biomolecules.

## 1 Introduction

The spontaneous organization of initially chaotic biological systems has helped scientists to explore the origins of Life on Earth.^1^, ^2^, ^3^, ^4^, ^5^ Indeed, it is widely accepted that the self‐assembling propensity of DNA, RNA, proteins, and peptides is one of the main molecular mechanisms that may have sparked Life as we know it. The so called “bottom‐up” approach to design self‐assembling materials, is directly inspired by this fascinating phenomenon.^6^, ^7^, ^8^

Self‐assembling materials are also used as models to study molecular mechanisms that cause abiogenesis and Alzheimer's disease.^1^, ^2^, ^3^, ^4^, ^5^ Moreover, self‐assembling peptides, inspired by the properties of biomolecules, have been developed for applications in diverse nanoscience sectors such as electronics, material science, and regenerative medicine.^9^, ^10^ The identification of stable domains that act as novel structuring motifs is critical for the development of self‐assembling biomaterials.^7^, ^8^, ^10^ Furthermore self‐assembling peptides (SAPs) are promising building blocks for tissue engineering due to their favorable biocompatibility, tailorability and biomimetic properties.^11^, ^12^, ^13^, ^14^ In the last decade our efforts have been focused on the development of SAP hydrogels for nervous regeneration and we designed different classes of SAPs such as functionalized SAPs,^15^ complementary coassembling peptides (CAPs) and BMHP1‐derived SAPs.^16^, ^17^ These peptides, featuring a promising pro‐regenerative potential in neural tissue engineering applications, form differently β‐structured filaments depending on their sequences and, in the case of BMHP1‐derived SAPs, depending on the presence of biotin at the N‐terminus.^17^, ^18^ Others demonstrated that, before assembling into nanoscaled filaments, SAPs initially self‐aggregate into oligomeric molten globules that are shaped by hydrophobic interactions.^19^, ^20^, ^21^ Within such molten globules peptides can adopt conformations of packing similar to paracrystalline and ordered nuclei. According to the protein nucleation mechanism this organization is due to the interplay between the forced spatial confinement of peptides and optimized (in terms of potential energy) nonbonded interactions.^22^ Smith and colleagues demonstrated that amyloid cross‐β structures of the peptide Aβ(16–22) can assemble through a dynamic conformational phylogeny. In their work isotope‐edited infrared spectra allowed to quantify the relative distribution of paracrystalline intermediates formed from intermolecular molten globules in which nucleation occurred previously.^19^

Furthermore, molecular dynamics simulations are a powerful tool to study peptide self‐assembly, and showed that molten peptide oligomers could act as incubators for β‐structuring.^23^, ^24^, ^25^, ^26^, ^27^, ^28^, ^29^, ^30^, ^31^, ^32^ Nonetheless, at the molecular level it is still unclear how oligomer‐to‐fibril transition emerges. Indeed, currently available tools to analyze molecular dynamics do not allow to track key events of the self‐assembling process such as the evolution of secondary structure patterns over time.

Coarse‐grained molecular dynamics (CG‐MD), enabling the simulation of larger systems on longer simulation times, showed great potential for high throughput screenings of the self‐assembling propensity of biomolecules. In addition, CG‐MD simulations allowed to estimate self‐assembling propensity of different peptide sequences for a wide latitude of potential applications.^33^, ^34^, ^35^, ^36^ Nonetheless, analytical tools for the quantitative tracking of secondary structure patterns (such as β‐structures) over MD trajectories are still lacking. This is an important limitation, given that knowledge of the time‐dependent formation of secondary structures is crucial for a deeper understanding of the self‐assembling phenomenon. In order to recognize β‐structuring domains, we developed a topological pattern recognition software based on the multilayer graph theory, named Morphoscanner, and we validated it on diverse protein structures. Here, we applied Morphoscanner to MARTINI CG‐MD simulations of peptide systems featuring spatial dimensions that are typical of molten particles.^1^, ^19^, ^20^ Simulations have been designed to mimic the experimental conditions that trigger peptides to self‐assemble into nanostructured hydrogels.

Thanks to the high adaptability of our software‐suite to different sequences and system sizes, we could demonstrate that SAPs exhibit sequence‐dependent intraoligomer organization in agreement with previously described self‐assembly models.^15^, ^16^, ^17^, ^18^, ^19^, ^20^

## 2 Results and Discussion

### 2.1 Morphoscanner

Network analysis and graph theory have found many applications in the study of protein structures and dynamics. Many studies focused on the development of algorithms for recognition or prediction of secondary structures such as α‐helix and β‐sheet arrangements. Due to of the large number of information to be taken into account (e.g., solvent accessibility, contact potentials, residue types),^37^, ^38^, ^39^, ^40^ however, the main application of the currently available algorithms consists in the analysis of single Protein Data Bank (PDB) structures. Indeed, many software packages cannot be applied to analyze molecular dynamics (MD) trajectories. Furthermore, currently available MD analysis tools for secondary structure analysis do not include a software adaptable to different coarsening levels of the simulated systems.^41^, ^42^, ^43^ To identify β‐sheet arrangements and to study their relative alignments in MD simulations we developed the Morphoscanner tool. The classical flat β‐sheet arrangement was first described by Pauling and Corey as a rectangular flat shape. However, crystallographic studies showed that β‐sheets tend to fold into saddle‐shaped surfaces as result of the interplay of individual peptide twisting and interchain hydrogen bonding. While the recognition of the hydrogen bond pattern is important for the identification of β‐structures, hydrogen bonds (H‐bonds) are sometimes not explicitly modeled in MD models. To identify both flat and twisted arrangements compatible with β‐sheets, Morphoscanner represents the peptide system as a 2D‐lattice graph defined on two axes:^42^, ^44^ one runs parallel to the backbone direction, the other one goes parallel to the H‐bonds direction. The edges along backbone direction represent covalent bonds. Instead, each edge along H‐bonds direction represents a β‐contact if the center‐of‐masses of two backbone group‐of‐atoms belonging to different peptides are closer than 4.7–5.3 Å, which is the typical inter β‐strand distance in β‐sheet structures (see Supporting Information).^45^ The β‐contacts network has been numerically represented trough a matrix (dubbed BB matrix) which is used to dynamically rationalize the global and local amount of the “in & out‐of‐register” mutual disposition of strands in the system.^46^ For this purpose, the BB matrix has been coarsened from residue‐to‐residue to strand‐to‐strand interaction level, thereby yielding a strand potential β‐interaction matrix named *P* matrix. Such “potential β‐interaction” is used to underline that the topological organizations identified by Morphoscanner are compatible with β‐structures. For sake of brevity “β‐interactions” will be used along the text instead of “potential β‐interactions.” The calculation of β‐interactions was achieved through a pattern‐matching algorithm comparing the BB matrix with a set of shift matrices describing all possible mutual alignment between two strands.^47^ Indeed, each shift matrix describes a single mutual alignment of two adjacent strands in function of the *k* parameter called shift value (see the Supporting Information for details) indicating their respective degree of sliding. For sake of clarity the possible parallel and antiparallel alignments (with positive or negative shifts) of the tested peptides are represented in Tables S6 and S7 of the Supporting Information. The dynamic reconstruction of topologies compatible with β‐structuring has been faced with a dynamic multilayer network approach.^48^ The interaction network formed by all residues in the system is investigated using the β‐contacts (BB matrix) and β‐interactions (*P* matrix), then the potential β‐structures are identified through the following heuristic: 1) a triplet of consecutively adjacent strands is identified in the P matrix, 2) and that same triplet has to satisfy in the BB matrix the minimum conditions for the number and distribution of inter‐strands H‐bonds. In brief, in a triplet of strands, the same portion of one strand must form a minimum of three β‐contacts with each of two neighboring strands. The iteration of such continuity criteria allows to identify both stable and evolving β‐sheets.

### 2.2 Morphoscanner Validation

The primary input for Morphoscanner is a series of contact map derived from PDB structures or molecular dynamics trajectories. Morphoscanner requires the following information: the number of strands (S) and the number of amino‐acid residues per strands (strand length, SL) in which the protein sequence should be divided. Morphoscanner returns different outputs and calculates the β‐strand percentage (%Ms) as follows

(1) $$\%\mathrm{Ms}=\frac{\mathrm{Number}\;\mathrm{of}\;\beta \mathrm{–strands}}{\mathrm{Number}\;\mathrm{of}\;\mathrm{strands}}*100$$

**Figure 1:**
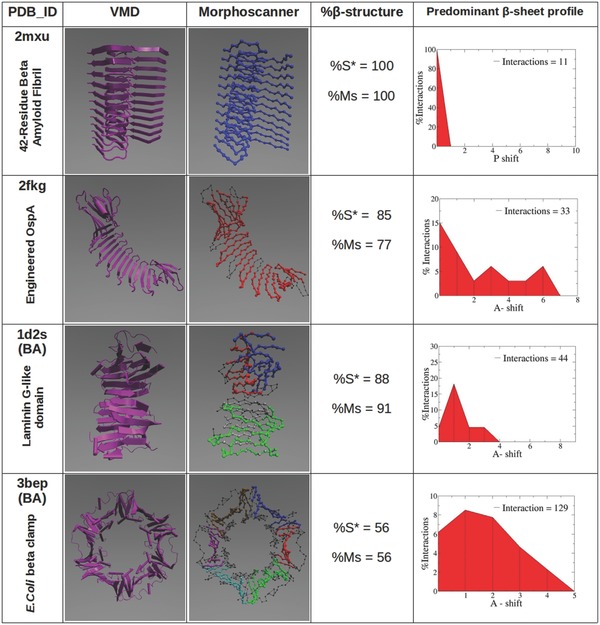
Morphoscanner validation on different protein structures. A series of PDB structures were analyzed with STRIDE‐based R script and Morphoscanner. In the first column, the reference PDB structures are represented as cartoon using VMD. In the second column, CG structures are visualized highlighting β‐sheets identified through Morphoscanner. β‐strand percentages calculated via R script (%S*) and Morphoscanner (%MS) are shown in the third column. In addition, shift profiles were used to quantify strand displacement in each structure. In the last column we depicted just the predominant shift profile. P = parallel alignment; A+ = antiparallel alignment with positive shift; A‐ = antiparallel alignment with negative shift. The analyses of 2mxu (SL = 32, S = 12) were in agreement and showed that strands were parallel aligned. In 2fkg (SL = 9, S = 35) strands were preferentially antiparallel aligned. The same conclusions were reached for 1d2s (SL = 10, S = 34) and 3bep (SL = 6, S = 122) analysis.

Also, we introduced an intuitive graphical representation called “shift profile” in order to highlight the preferential arrangement of strands. To validate Morphoscanner we analyzed some protein structures as reported in **Figure** **1**. As our main focus was to characterize coarse‐grained systems of SAPs, protein structures from PDB were CG‐mapped according to the MARTINI model and subsequently analyzed using Morphoscanner (given the intrinsic versatility of Morphoscanner, this procedure could have been performed on other levels of structural coarsening). Thanks to web server STRIDE, the secondary structures assignment for each PDB structure could be readily computed. The STRIDE output files were evaluated through an in‐house developed “R script” which works similarly to Morphoscanner. The above cited script returns the β‐strand percentage (%S*), similarly to the Morphoscanner output

(2) $$\%S^{*}=\frac{\mathrm{Number}\;\mathrm{of}\;\beta \mathrm{–strands}}{\mathrm{Number}\;\mathrm{of}\;\mathrm{strands}}*100$$

The comparison between %S* and %Ms was used to determine if Morphoscanner successfully identified β‐sheet structures.

Amyloid plaques are a defining characteristic of Alzheimer's disease. The Aβ(1‐42) fibrils is the initial and predominant constituent of amyloid plaques. We investigated the Aβ(1‐42) amyloid fibrils (2mxu) structure. To analyze this structure, SL and S were set to values of 32 and 12, respectively. The ssNMR analyses of Aβ(1‐42) amyloid fibrils revealed parallel β‐strands arrangement.^49^ The Morphoscanner analyses were fully in agreement with the abovementioned structural investigations, and they were confirmed by a STRIDE analysis of PDB structures (%S* = 100, %Ms = 100), as shown in Figure 1. Indeed, all the potential β‐sheet structures showed a preferential parallel in‐register alignment.

We further tested Morphoscanner on an engineered *Boriella* OspA structure (2fkg), a β‐sheet rich self‐assembly mimicry. Its structure consists of β‐hairpin repeats connected by turn motifs.^50^ To analyze the 2fkg structures, SL and S were set to 9 and 35, respectively. The analyses through STRIDE and Morphoscanner provided similar results (%S* = 85, %Ms = 77). In agreement with structural analysis performed by Makabe et al., Morphoscanner identified an antiparallel out‐of‐register β‐strands organization (see Figure 1; Figure S1, Supporting Information). In addition, β‐sheet profiles revealed a different progressive strand displacement accountable by twisting and bending between different strands.

Morphoscanner was also tested on different biological assemblies that have pivotal roles in diverse metabolic pathways. Laminin are high‐molecular weight proteins of the extracellular matrix and constitute the biologically active part of the basal lamina, influencing cell differentiation, migration, and adhesion. These proteins consist of different subunits comprising the lamin‐g‐like module (see 1d2s in Figure 1) which mediates the binding to different molecules such as heparin and the cell surface receptor alpha‐dystroglycan (alpha‐DG).^51^ To perform the analysis of the 1d2s structure, SL and S were set to 10 and 34, respectively. The Morphoscanner and STRIDE analyses reported the same results (%S* = 88, %MS = 91). In addition, as demonstrated by crystallographic analyses, Morphoscanner revealed a preferential antiparallel alignment among potential β‐strands (see Figure S1, Supporting Information).

*Escherichia coli* beta clamp is a subunit of the DNA polymerase III holoenzime which consists of two identical subunits, made of 366 residues each.^52^ To obtain a periodic division of 3bep structure, SL and S were set to 6 and 122, respectively. Morphoscanner identified the antiparallel β‐sheet structures, as shown in Figure 1; Figure S1 (Supporting Information), which could be perfectly superimposed to β‐sheet representation obtained by VMD. This was also demonstrated by comparison between Morphoscanner and STRIDE statistics (%S* = 56, %Ms = 56), as shown in Figure 1.

### 2.3 Modeling of Assembling Systems: BMHP1‐Derived SAPs, CAPs, and (LDLK)_3_

Looking for a broad SAP analysis, CG‐MD simulations were used to study the self‐assembling propensity of seven punctually mutated BMHP1‐derived SAPs,^18^ the almost neutral (LDLK)_3_ SAP,^53^ and the two complementary charged (LDLD)_3_ + (LKLK)_3_ CAPs (see **Table** **1**).^16^ Systems comprised a total of identical 100 peptides for BMHP1‐derived SAPs and (LDLK)3, and 50 plus 50 oppositely charged peptides in case of mixed CAPs.

**Table 1:** All BMHP1‐derived SAPs (2,B3,4,B24,B26,30,31) systems were simulated at 3% (w/v) to mimic the standard empirical conditions enabling nanostructured hydrogel formation. (LDLK)_3_ and CAP concentration was 1% (w/v). SS parameters of all residues were set to extend. Three CGMD simulations have been carried out for each system up to 500 ns: one simulation per each set was prolonged to 2000 ns. Lastly, simulations were further prolonged to 4500 ns for (LDLK)_3_, (LDLD)_3_ + (LKLK)_3_, B24 and 30

Sequence ID	Sequence	Box size [nm]	CG ions beads (NA+/CL−)	CG Water beads	N° of peptides	N° sim x time [ns]
2	GGGPFSSTKT	17.56	50/150	43770	100	1 × 2000
						2 × 500
B3	Btn‐GGGPFSSTKT	18.6	60/160	52213	100	1 × 2000
						2 × 500
4	WGGGPFSSTKT	18.61	60/160	52480	100	1 × 2000
						2 × 500
B24	Btn‐GGGAFASTKT	18.35	58/158	50314	100	1 × 4500
						2 × 500
B26	Btn‐GGGPFASTKT	18.52	59/159	51038	100	1 × 2000
						2 × 500
30	WGGGAFASTKT	18.4	58/158	50311	100	1 × 4500
						2 × 500
31	WGGGAFSSTKT	18.42	58/158	50612	100	1 × 2000
						2 × 500
(LDLK)_3_	LDLKLDLKLDLK	28.8	0/0	200058	100	3 × 4500
(LDLD)_3_+ (LKLK)_3_	LDLDLDLDLDLD+LKLKLKLKLKLK	28.8	184/184	199649	50 + 50	3 × 4500

In this work molecular interactions in CG‐MD simulations were modeled by the MARTINI force field that has recently showed promising potential for the high‐throughput screenings of SAPs.^54^, ^55^, ^56^ In MARTINI, four heavy atoms are usually represented by one CG bead, while a lower ratio is used for atoms involved in rings. Bonded interactions are described with bond, angle and dihedral energy functions, while nonbonded interactions are described through Lennard‐Jones and Coulomb functions.^55^, ^56^, ^57^, ^58^ Given that some of the BMHP1‐derived SAPs include N‐terminal Biotin‐tag, which was not yet available for the MARTINI force‐field, we parametrized the biotin tag as follows: structural and interaction parameters were extrapolated from previous UA simulations and validated through octanol/water partition coefficient (logP) calculations (see the Supporting Information for details).^18^, ^54^, ^55^ Experimental and calculated logP values did not show significant differences.

Notably, in MARTINI the secondary structure of molecules is fixed throughout the simulation, therefore the choice of the secondary structure (SS) parameters is crucial for the reliability of the modeled system. A fully extended secondary structure was adopted for both (LDLK)_3_ and CAPs because of 1) the presence of equally spaced identical or opposite charges along the same short peptides and 2) their typical β‐sheet signature in circular dichroism spectra.^16^, ^53^

In case of BMHP1‐derived SAPs, in line with previously published works,^15^, ^17^, ^18^, ^59^ the chosen secondary structure assignment was initially derived by comparing united atom (UA) and CG simulations of octameric systems. In CG simulations of octamers, three different secondary structure sets (all extended, all coil or sampled conformational distribution of monomers in UA simulations) were combined with two starting structures distributions: all extended and sampled configurations of monomers in UA. After comparing gyration radius, aggregation order, and alignment degree (see the Experimental Section for details and Figures S2–S4, Supporting Information) of UA and CG simulations of octamers, we chose fully extended secondary structures and sampled structural configurations for the subsequent CG simulations of BMHP1‐derived peptides 100‐mer systems.^55^

### 2.4 Using Morphoscanner for the Analysis of Self‐Assembled Peptidic Aggregates

**Figure 2:**
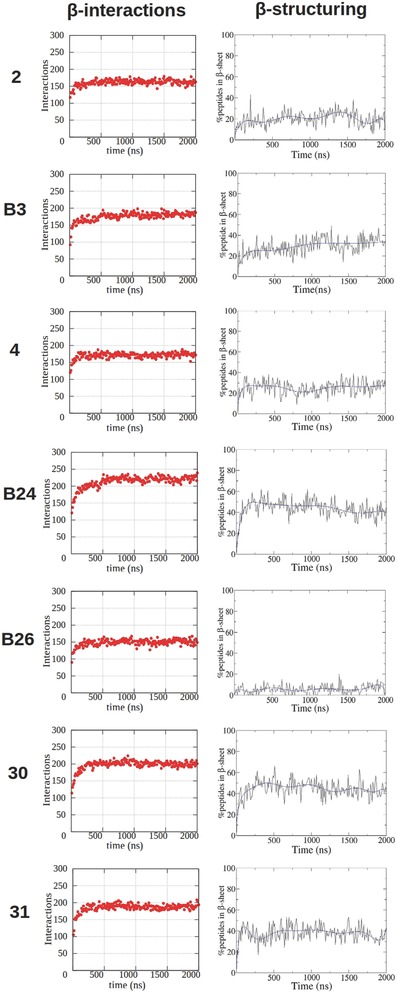
β‐interactions and β‐structuring of SAPs in CG‐MD simulations with extended SS parameters. The onset of β‐interactions does not warrant the formation of β‐sheet structures. This is clearly evident from the comparison among peptides 2,4 and B26. The above‐mentioned SAPs reached the same number of β‐interactions, but B26 had the lowest degree of β‐structuring propensity, followed by 2 and 4. Such features are attributable to their sequences and, in particular, to *N*‐terminal functionalization.

**Figure 3:**
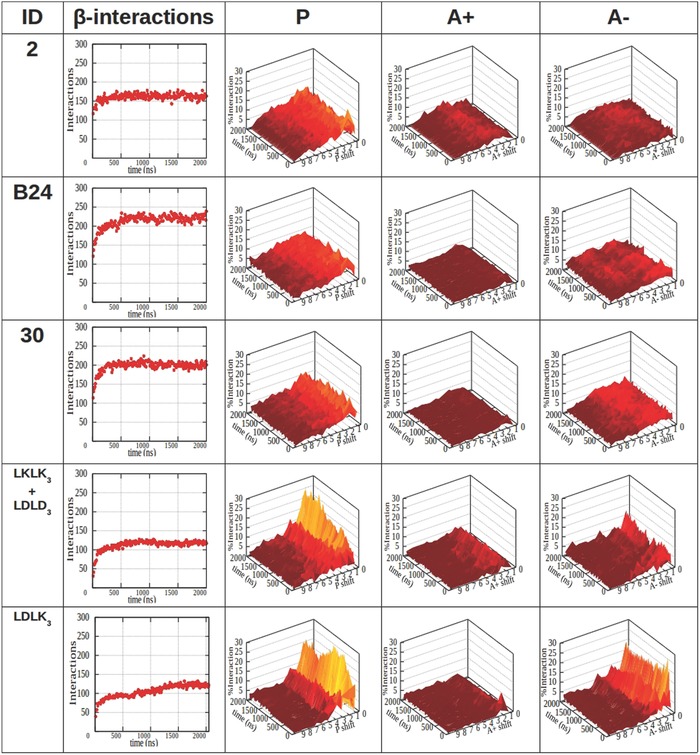
Analysis of mutual alignment of peptides featuring diverse self‐assembling propensities. Peptides mutual alignment shift profiles of SAP 2, B24, and 30 which were simulated with extended secondary structure parameters (see Table 1). P refers to parallel alignment, A+ to antiparallel alignment with positive shift, A‐ to antiparallel alignment with negative shift. BMHP1‐derived SAPs preferentially shifted by one residue in P alignment, but (LDLK)_3_ and CAPs showed much stronger alignment in both P and A‐ alignments at one residue shift. This feature was likely due to the electrostatic interactions among their complementary charged side‐chains. On the other hand, the mutation of Pro and Ser with Ala increased the number of β‐interactions in B24 and 30 assemblies if compared to SAP 2 (see Table 1). Biotinylation also slightly improved β‐sheet structuration propensity in B24 in respect to 30. Notably, CAPs and (LDLK)_3_ showed less β‐interactions than BMHP1‐derived SAPs. This was due to the different shapes of supramolecular aggregates; (LDLK)_3_ and CAPS formed bilayered β‐sheet‐rich aggregates. BMHP1‐derived SAPs formed ovoid aggregates where peptide strands could simultaneously interact with multiple surrounding peptides.

In MARTINI CG‐MD simulations, the fixed SS parameters allow to discriminate between various secondary structures, however, they do not allow to detect any secondary structure transitions. Notwithstanding this limitation, it is possible to evaluate the movement of secondary structure elements in the simulated systems.^54^, ^55^ Morphoscanner was used for the analyses of the CG‐MD simulations of SAPs in Table 1 with extended secondary structure parameters. S and SL parameters were set equal to the number of peptides and of backbone grains per peptide, respectively. The organization of the simulated systems over time was schematized into a count of both total the β‐interactions in the system and the percentage of peptides taking part in potential β‐sheets formation (**Figures** **2** and **3**). We used a shift profile representation over time (Figures 2 and 3) to track peptides preferential arrangement during self‐assembling. Lastly, the shift profile approach was adopted to monitor peptides arrangement within β‐sheets structures (Figure S5, Supporting Information).

The total number of β‐interactions in BMHP1‐derived SAPs, CAPs, and (LDLK)_3_ was 150 to 240. B24 showed the highest number β‐interactions, while the lowest numbers were found in CAPs and (LDLK)_3_ (Figure 3; Figure S5, Supporting Information), caused by different peptides arrangement within oligomers. CAPs and (LDLK)_3_ assembled into bilayered structures made of peptides packed side‐by‐side. Instead peptide B24, similarly to other BMHP1‐derived SAPs, assembled in ovoid oligomers where interactions among neighboring peptides were favored.

BMHP1‐derived SAPs preferentially aligned in parallel out‐of‐register of one residue (≈10–15% of total β‐interactions). CAPs and (LDLK)_3_ were preferentially shifted by one residue in P (≈10%) and in A‐ alignments (≈25%). As shown in Figure 3 and Figure S5 (Supporting Information), all the potential β‐interactions in aggregates formed by CAPs took part in β‐sheet structures. Indeed, all peptides contributed to β‐sheet formation in all simulations within 50 ns (data not shown).

**Figure 4:**
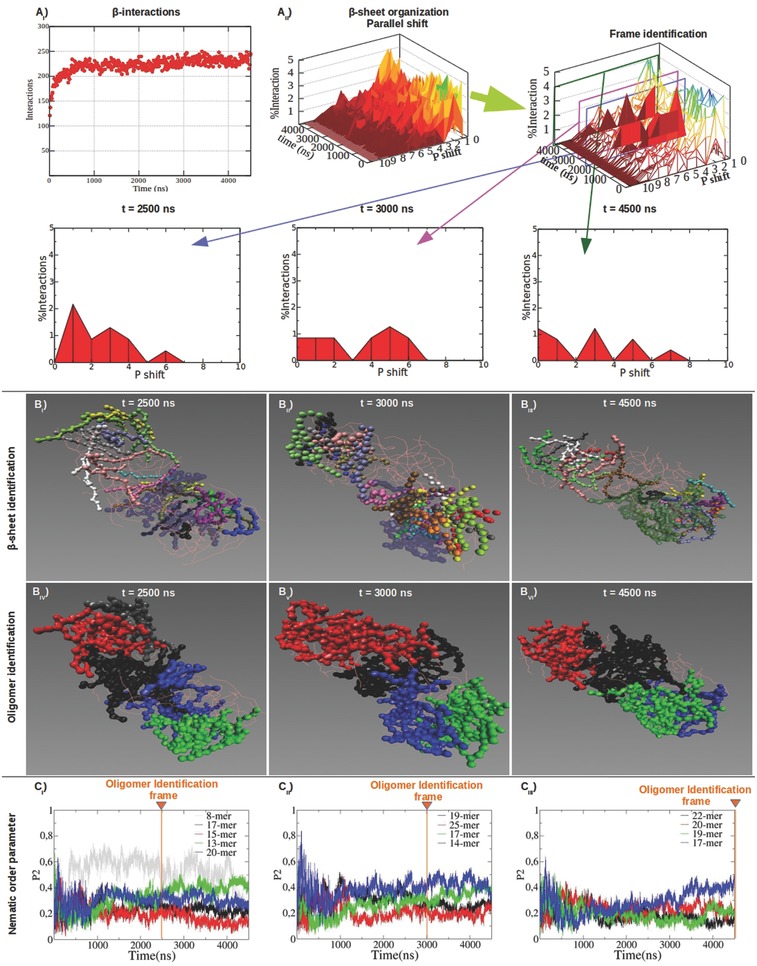
Structural characterization of B24 molten particles at different timeframes. B24 showed good β‐sheet propensity (A_I_) characterized by parallel out‐of‐register β‐strands (A_II_). Parallel β‐sheets shift profiles became wider between 2500 and 4500 ns: this was matched by changings in β‐sheet topology (B_I–III_) and influenced the identification of oligomers (B_IV–VI_). P2 was calculated for the identified oligomers (C_I–III_). Same colors between B_IV–VI_ and C_I–III_ point at the same oligomers identified at the selected timeframes. P2 values of the identified oligomers were calculated for all timeframes. The identified oligomers ranged from 8‐mer to 25‐mer aggregates. Interestingly, oligomers (B_IV–VI_) featuring higher order (or P2 values in C_I–III_) showed a large presence of β‐sheets (B_I–III_).

BMHP1‐derived peptides generally showed a variable β‐structuring propensity related to the punctual mutations in their sequences (see Table 1). SAP 2, made of the BMHP1 motif and a triplet of Gly, did not show a good β‐structuring propensity. Only 10% of the total simulated SAP 2 peptides took part in β‐sheets formation (Figure S1, Supporting Information) and they preferentially aligned in parallel out‐of‐register by 1 residue (Figure 4; Figures S6 and S7, the Supporting Information**)**. The introduction of Trp at the N‐terminus improved the β‐structuring propensity of SAP 4, with 25% of peptides involved in β‐structuring (Figure S5, Supporting Information). Biotinylation increased the β‐sheet structuring propensity in B3: indeed 30% of peptides were involved in β‐sheets structures (Figure 2). B24 showed the highest propensity to β‐sheet structuring. Indeed, 50% of B24 peptides fell within β‐sheets (Figure 2), and the large part of pairs of β‐strands were preferentially aligned in parallel out‐of‐register with neighboring pairs by one and two residues as shown in Figure 1 and Figure S7 (Supporting Information). These increments were ascribable to both the N‐terminal biotinylation and the substitution of Pro and Ser with Ala. The substitution of Btn with Trp decreased the formation of stable β‐sheet structures in peptide 30 in comparison with B24 (Figure 2): even if they showed similar preferential alignments (Figure 3; Figure S7, Supporting Information) just 40% of peptides were involved in β‐sheet structures. In the case of peptide 31 the first Ser of the BMHP1 motif was mutated with Ala, but apparently, when compared to SAP 30, did not alter the β‐structuring propensity of the system (Figure 2). The introduction of biotin at the N‐terminal position did not improve β‐structuring propensity in B26 (Figure 2): only 10% of peptides were part of β‐sheets. As shown in Figures S6 and S7 of the Supporting Information B26 peptides were preferentially aligned in parallel out‐of‐register by one residue and nine residues, likely because of preferential pairings between Lys backbone and Biotin amide groups.^60^, ^61^

### 2.5 Peptide Oligomer Identification

To more efficiently describe the onset and subsequent arrangement of “seeds of self‐assembling” within the molten globules we combined our recently introduced methodology with classical analyses, such as radius of gyration and nematic order parameter.^42^ However, in big system simulations locally ordered aggregates may not be described by cumulative parameters of the overall system: therefore, it was necessary to track the oligomers formed during the self‐assembling process. Indeed, others proposed a nucleation‐dependent polymerization model to describe fibril formation from monomeric peptides to heterogeneous nuclei (or peptide micelles) and finally mature fibrils.^62^, ^63^, ^64^ Oligomer identification required the implementation of a “nearest neighbor algorithm” on binary entries obtained by thresholding distances among peptide center‐of‐masses. Threshold distance was set at 1.1 nm as in XRD spectra it represents the typical equatorial distance of cross‐β structures.^64^ In the so‐obtained contact matrix 1 value points at a distance falling within the chosen range, thus giving a “forest” of ones.^65^ The algorithm first explores each subtree representing neighboring center‐of‐masses, then backtracks and provides the peptides constituting each oligomer.^66^

### 2.6 Tracking Oligomers Arrangement Dynamics and β‐Structures Organization

The combined use of the Morphoscanner and of the oligomer identifier algorithm allowed to detect oligomeric species with different local structural features. Then, we obtained a more detailed analysis of the β‐structuring propensity of the simulated systems. As previously mentioned peptide B24 displayed good β‐structuring propensity (Figure 2) and the total amount of β‐interactions stabilized after 500 ns. However, **Figure** **4**A_II_ shows that β‐sheets changed their internal organization (see also Figure S8, Supporting Information); at 2500 ns β‐strands were preferentially aligned in parallel out‐of‐register by one residue, but this was not the case at 3000 ns and at 4500 ns. Oligomers showed limited variations in global morphology and mutual disposition (Figure 4B_IV–VI_) over time. As shown in Figure 4C, the nematic order parameter P2 of almost all identified oligomers at different time‐points fluctuated below 0.5: this was indicative of a modest (but still developing) peptide arrangement over time.

**Figure 5:**
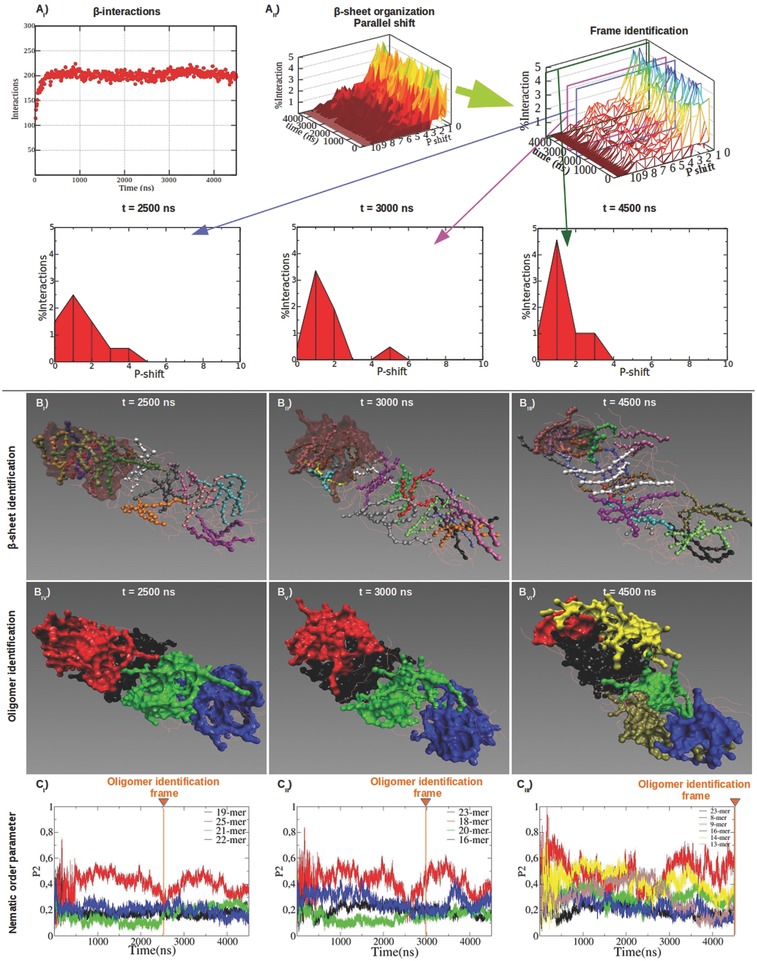
Structural characterization of 30 molten particles at different timeframes. SAP 30 had a good β‐structuring propensity (A_I_) and peptides were mutually aligned in parallel out‐of‐register by one residue within β‐sheets (A_II_). Shift profiles of parallel β‐sheets became sharper after 2500 ns but did not vary as extensively as in B24. The topology of β‐sheets changed slightly (B_I–III_): this was reflected in a modest variation of the identified oligomers at different timeframes (B_IV–VI_). Same colours between B_IV–VI_ and C_I–III_ point at the same oligomers identified at the selected timeframes. P2 values of the identified oligomers were calculated for all timeframes. More ordered oligomers (C_I–III_) were characterized by stronger presence β‐sheet structures (B_I–III_). The oligomers identified at 4500 ns were more heterogeneous and with higher P2 values (C_I–III_): big oligomers identified in previous timeframes were here split in two or more subgroups.

The same analysis workflow was applied to peptide 30. In agreement with previous empirical studies,^17^ the mutation of Biotin with Trp in peptide 30 yielded to a more stable β‐structuring (**Figure** **5**; Figure S9, Supporting Information) than in B24. Indeed, from the early stage of SAP 30 self‐assembling, β‐strands were preferentially aligned in parallel out‐of‐register by one residue (Figure 5A; Figure S9, Supporting Information). In addition, more similar oligomers of peptide 30 were identified at different time‐points (Figure 5B_IV–VI_) and their calculated order was mostly higher than in B24 (Figure 5C).

**Figure 6:**
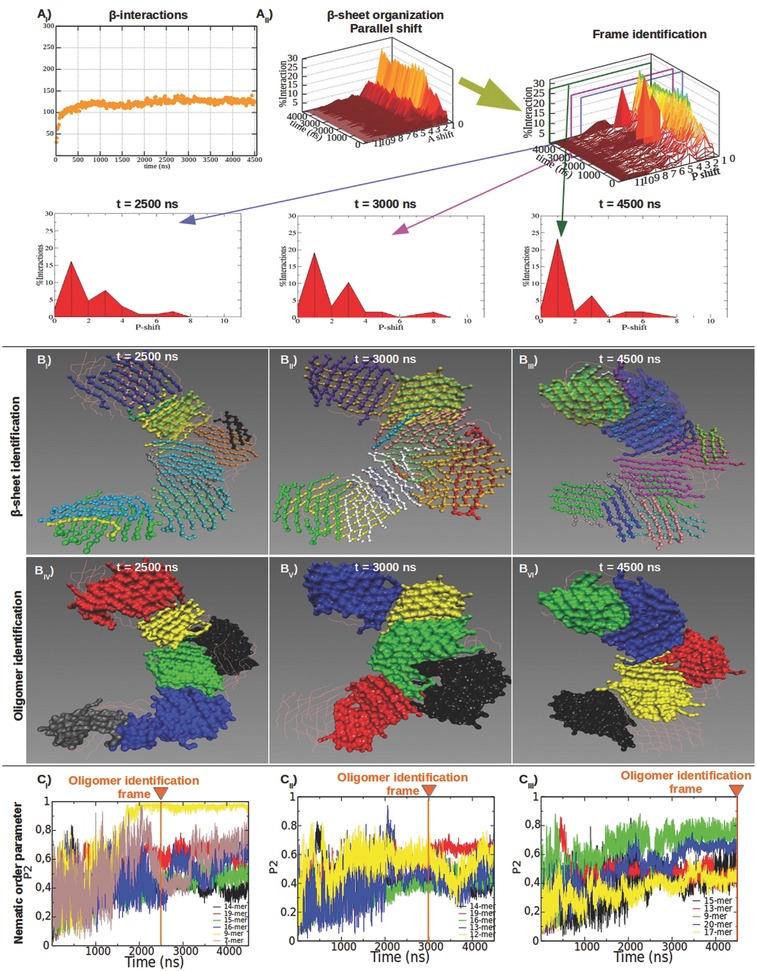
Structural characterization of CAPs (LDLD)_3_ + (LKLK)_3_ molten particles at different timeframes. CAPs established less ß‐interactions (A_I_) than in BMHP1‐derived SAPs and β‐strands were preferentially aligned in parallel out‐of‐register by one residue throughout the simulations (A_II_). CAPs formed stable β‐sheet structures (B_I–III_) mainly matching oligomers distribution (B_IV–VI_). β‐sheets paired into bilayered aggregates but with different orientations. Same colours between B_IV–VI_ and C_I–III_ point at the same oligomers identified at the selected timeframes. P2 values of the identified oligomers were calculated for all timeframes. The identified oligomers displayed a superior order (values of P2) and a slow but ongoing trend of increments toward more ordered assemblies (C_I–III_).

CAPs, thanks to their alternated opposite charged side‐chains, self‐assembled into stable β‐sheets (**Figure** **6**A), forming a “patchwork” of bilayered aggregates.^16^ CAPs formed bilayered oligomers (Figure 6B_IV–VI_) characterized by a significant presence of β‐sheets (Figure 6B_I–III_) and by an high internal alignment (Figure 6C). This behavior was dictated by strong electrostatic interactions among oppositely charged side‐chains fostering the formation of well‐defined β‐structures. Nonetheless, variable oligomer identification and unstable P2 values revealed a still ongoing arrangement of the system given by a persistent “sliding” of the patches composing the two layers. The same tendencies were also observed for LDLK_3_ peptides. Indeed, as reported in our previous work, such peptides formed β‐structured and highly ordered bilayered aggregates.^67^

## 3 Conclusions

Morphoscanner, a novel software developed for secondary structure analysis of differently coarsened simulations of proteinaceous structures, combines into a single “suite” the advantages of both MD analysis and secondary structures pattern recognition tools.^39^, ^40^, ^41^, ^42^ On previously characterized protein structures Morphoscanner recognized their β‐sheet organization and provided new information about their relative orientation and alignment. In Morphoscanner we also included a new high‐throughput workflow to investigate different facets of self‐assembly: its graphical and quantitative analyses provided new insights of SAP systems evolution over time. It was possible to more efficiently elucidate the self‐assembly process of BMHP1‐derived peptides, (LDLK)_3_, and CAPs. BMHP1‐derived SAPs self‐assembled into molten particles mostly composed of peptides aligned in parallel out‐of‐register, a thermodynamically stable alignment that however may prevent any subsequent evolution toward well‐structured nanofibers detected in previous experimental works.^1^, ^17^, ^19^ On the other hand, (LDLK)_3_ and CAPs formed patches of anti‐parallel β‐rich aggregates evolving toward cross‐β packings, yielding to highly ordered systems compatible (at longer timeframes) with empirical observations.^1^, ^16^, ^17^

Lastly, we developed a software suite useful for the analysis of molecular assembly, easily adaptable to other chemical species and coarsening levels. Indeed it can be potentially applied to the study of biological processes such as DNA, RNA hybridization or abnormal protein assembly.^62^, ^68^, ^69^ Thus, the achieved level of characterization may turn useful in nanotechnology but also in biomolecular and astrobiological studies focused on the emerging properties of self‐assembling systems.^2^, ^3^, ^4^, ^5^, ^6^

## 4 Experimental Section

*MD of BMHP1‐Derived SAPs*: The sequences of the simulated BMHP1‐derived SAPs are listed in Table 1. Peptide monomers have the C‐terminus amidated and the N‐terminus biotynilated (or acetylated). Lysine residues are in the protonated state. Extended conformations of monomers were built with Pymol software by imposing all‐trans geometry on the backbone dihedrals. Molecular dynamics were run using version 4.5.5 of the GROMACS simulation package and the GROMOS53a6 force field: systems comprised eight monomers each as reported previously and explicit aqueous solvent.^18^ Coarse‐grained molecular dynamics simulations have been conducted on octameric or 100‐meric systems using MARTINI force field version 2.2. The choice of secondary structure parameters for 100‐meric systems was made by comparing UA‐MD and CG‐MD simulations of 8‐meric systems of BMHP1‐derived SAPs.

*Choosing the Secondary Structure Parameters in CG‐MD Simulations of BMHP1‐Derived SAPs*: To select the most appropriate secondary structure parameters for 100‐mer system simulations, UA and CG simulations of 8‐mer systems were compared. Gyration radius, nematic order parameter, and the aggregation curves were used to assess the agreement between UA and CG molecular models.^18^ Starting configurations of the systems modeled in UA and CG simulations consisted of extended (*E*) or UA sampled (SAM) monomers comprising the octameric systems. Three different choices of SS parameters were proposed in CG‐MD simulations: fully extended, coil or UA‐sampled secondary structures (see Tables S8–S10, Supporting Information). The SAM secondary structures parameters are monitored on UA‐MD simulations by means of the DSSP algorithm as reported in the previous work.^18^ CG‐MD simulations (using the abovementioned sets of SS parameters) and UA‐MD simulations were then compared. CG‐MD simulations of UA‐sampled conformers with fully extended SS parameters resulted in gyration radii, nematic order parameters, and aggregation orders in higher agreement with the UA simulations, as shown in Figures S2–S4 of the Supporting information.

*CG‐MD Simulations of 100‐mer Systems of BMHP1‐Derived SAPs*: The boxes containing unsolvated peptides were built using the PACKMOL software.^70^ UA‐sampled monomer conformations were inserted in random orientations and positions, so that the atoms belonging to different peptides were at least at 10 Å away from each other.^18^ Boxes, filled with MARTINI CG water beads, were chosen so as to mimic the 3% (w/v) concentration of SAPs typically used in empirical tests. As mentioned in Section 2.3 fully extended SS parameters were adopted (see Table 1). Ions (Na+ and Cl−) were added to neutralize the systems up to 0.015 m concentration of NaCl, in order to reproduce salt concentration of diluted PBS (1x) solution. The production phase was conducted using constant temperature, pressure, and number of molecules (i.e., the NPT ensemble). Temperature, pressure, constraints, cut‐off value, periodic boundary conditions, and integration‐step settings were identical to 8‐mer systems simulations. Three random distributions of peptides and ions were generated and simulated for 500 ns. One of the replicas as per each SAP sequence was extended up to 2000 or 4500 ns.

*CG‐MD Simulations of 100‐mer Systems of (LDLK)_3_ and (LDLD)_3_+(LKLK)_3_*: A similar approach was adopted for simulations of (LDLK)_3_ and CAPs. All‐trans configuration of the (LDLK)_3_, (LDLD)_3_, and (LKLK)_3_ were generated by Pymol (http://www.pymol.org/). The C‐ and N‐termini of peptide monomers were amidated and acetylated, respectively. At neutral pH, lysine and aspartic acid side chains, because of their weak basic and acidic nature, can be considered fully protonated and deprotonated, respectively. Peptide were distributed (using PACKMOL) in explicit water cubic boxes. Prior to production, systems underwent an equilibration phase (a 3000‐steps minimization using steepest descent method). The production phase was conducted in NPT ensemble in order to reproduce experimental conditions used in previous works.^17^, ^67^

*Strand/Peptide Alignment Analysis via Morphoscanner*: Despite the structural differences observed in crystallography, β‐sheets can be described as a regular 2D lattice graph stabilized by covalent bonds (along the direction of the backbone chains) and by hydrogen‐bonds (among the backbone chains). As previously mentioned, we introduced the definition of β‐contact to define the “edges” along H‐bonds direction (Equation (3))

(3) $$\beta -\;{\mathrm{contact}}_{ij}\;=\;\delta \left(r_{ij}-r_{0}\right)$$

where δ is the Dirac measure,^71^ *r_ij_* is the distance between backbone atom‐group (grain) center‐of‐masses *i* and *j*, *r*_0_ represents the distance between two β‐strands in cross‐β structures (range between 4.7 and 5.3 Å).^45^

The numerical representation of β‐contacts network is provided by the BB matrix, whose elements are described in Equation (4)

(4) $${\mathrm{BB}}_{ij}\;=\;\beta -\mathrm{contac}t_{ij}$$

The description of the interactions between two strands or peptides is provided by the Strand Backbone Contact matrix: a square matrix whose dimensions correspond to the number of strand/peptide backbone grains.

A set of matrices, named shift matrices, was developed to be used as references for the identification of the mutual arrangements described by the strand backbone contact matrix. Shift matrices describe the different arrangements between pairs of peptides.

In detail, parallel and antiparallel shift arrangements are described using the following matrix notation:

(5) $$\mathrm{Positive}\;\mathrm{shift}\;\mathrm{parallel}\;\mathrm{arrangement}\;\to P_{ij}^{+}=\delta_{i+k,j}$$

(6) $$\mathrm{Negative}\;\mathrm{shift}\;\mathrm{parallel}\;\mathrm{arrangement}\;\to \;P_{ij}^{-}=\delta_{i-k,j}$$

(7) $$\mathrm{Positive}\;\mathrm{shift}\;\mathrm{anti}-\mathrm{parallel}\;\mathrm{arrangement}\;\to A_{ij}^{+}=\delta_{n-i+k,j}$$

(8) $$\mathrm{Negative}\;\mathrm{shift}\;\mathrm{anti}-\mathrm{parallel}\;\mathrm{arrangement}\to A_{ij}^{-}=\delta_{n-i-k,j}$$

In the previous formulas *δ_ij_* identify the Kronecker delta, *n* is the number of peptide backbone grains, *i,j* are the indexes of peptide backbone grains (varying within *n*), and *k* is the shift value.

This set of matrices describing the peptide interaction library can be represented using the following compact notation:

(9) $$L=L_{ijz}$$

where *z* is the index of shift matrices in the library.

To calculate the maximum similarity of shift matrices with peptide backbone matrix, the normalized cross‐correlation function (NCC) was used^71^

(10) $$\mathrm{NCC}\;\left(p,q,z\right)=\frac{\sum_{i=0}^{\mathrm{RES}-1}\sum_{j=0}^{\mathrm{RES}-1}\mathrm{BB}\left(i+p*\mathrm{RES},j+q*\mathrm{RES}\right)*L\left(i,j,z\right)}{\sqrt{\sum_{i=0}^{\mathrm{RES}-1}\sum_{j=0}^{\mathrm{RES}-1}\mathrm{BB}\left(i+p*\mathrm{RES},j+q*\mathrm{RES}\right)}*\sqrt{\sum_{i=0}^{\mathrm{RES}-1}\sum_{j=0}^{\mathrm{RES}-1}L\left(i,j,z\right)}}$$

*Z* is the index of the shift matrix *L_ijz_* that maximize the value of NCC function. *P* and *q* denote the area of the BB matrix corresponding to the peptide backbone contact matrix.

Each element of the strand potential β‐interaction matrix, describing mutual alignment between couples of strands, is defined as follows

(11) $$P_{pq}=Z$$

*β‐Sheet Reconstruction via Morphoscanner*: Flat and twisted β‐sheet structures are detected by Morphoscanner by using the backbone contact and the strand interaction matrices, i.e., handling the peptidic system as 2D‐lattice graph. In detail, the algorithm identifies a triplet of strands making a β‐structure. It calculates the area of the system backbone contact matrix, corresponding to the interaction between the first pair of strands, and reduces this area to a row vector, as shown below

(12) $$v_{r}=\left(\sum_{i=0}^{n}\left(A\left(i,1\right)\right),\sum_{i=0}^{n}\left(A\left(i,2\right)\right),\ldots ,\sum_{i=0}^{n}\left(A\left(i,n\right)\right)\right)$$

Morphoscanner identifies the area corresponding to the other pair of strands, giving another column vector

(13) $$v_{c}=\left(\sum_{j=0}^{n}\left(A\left(1,j\right)\right),\sum_{j=0}^{n}\left(A\left(2,j\right)\right),\ldots ,\sum_{j=0}^{n}\left(A\left(n,j\right)\right)\right)$$

Finally, the projection of *v* **_r_** on *v* **_c_** is calculated as a dot product

(14) $$v_{p}=v_{r}*v_{c}=\left(v_{r1}*v_{c1},\;v_{r2}*v_{c2},\ldots ,v_{\mathrm{rn}}*v_{\mathrm{cn}}\right)$$

The number of consecutive residues defining a structuring β‐sheet along the covalent bonds direction is calculated as the maximum number of elements included between two non‐null elements. In this way, β‐sheet structures are identified as curved rectangular 2D‐lattices whose dimensions are defined by strands and by the number of backbone grains.

## Conflict of Interest

The authors declare no conflict of interest.

## Supporting information

Supplementary
