# Supplementary material for: Elucidating Self‐Assembling Peptide Aggregation via Morphoscanner: A New Tool for Protein‐Peptide Structural Characterization
Source: Adv Sci (Weinh). 2018 Jun 22;5(8):1800471. doi: 10.1002/advs.201800471 (PMC6097002; doi:10.1002/advs.201800471)
Supplement: Supplementary file 1 — Supplementary [file ADVS-5-1800471-s001.pdf]

Copyright WILEY-VCH Verlag GmbH & Co. KGaA, 69469 Weinheim, Germany, 2018.

ADVANCED SCIENCE Open Access

Supporting Information

for *Adv. Sci.,* DOI: 10.1002/advs.201800471

Elucidating Self-Assembling Peptide Aggregation via Morphoscanner: A New Tool for Protein-Peptide Structural Characterization

*Gloria A. A. Saracino, Federico Fontana, Shehrazade Jekhmane, João Medeiros Silva, Markus Weingarth, and Fabrizio Gelain**

Copyright WILEY-VCH Verlag GmbH & Co. KGaA, 69469 Weinheim, Germany, 2016.

Supporting Information

Elucidating self-assembling peptide aggregation via Morphoscanner: a new tool for protein-peptide structural characterization

Gloria A. A. Saracino^, Federico Fontana^, Shehrazade Jekhmane, Joao Medeiros Silva, Markus Weingarth and Fabrizio Gelain*

Supplementary Methods & Tables

CG-MD of octameric systems of BMHP1-derived SAPs

Initial conformations of the monomers were extrapolated by the conformational sampling of the single monomer from UA-MD simulations [1]. Initial configurations of the systems were prepared by insertion of the selected centro-types in random positions inside cubic boxes filled with MARTINI CG water-beads. Box sizes were chosen so as to reproduce the 1% (w/v) or (3% w/v) concentrations of SAPs used in previous empirical tests. Cl- and Na+ ions were added, up to the concentration of 0.015 M NaCl, to neutralize the charges of the solvated systems. Systems underwent an equilibration phase, i.e. a short-energy minimization step using the steepest descent method. The production phase was conducted using constant temperature, pressure and number of molecules (i.e. the NPT ensemble). Temperature was set to 300 K, by means of the coupling algorithm v-rescale (τ_t = 0.1 ps). Pressure was maintained at 1 bar, by means of the pressure coupling algorithm of Berendsen (τ_p = 0.1 ps). The integration step was set to 20 fs, while snapshots of individual trajectories were saved every 100 ps. Each simulation lasted 250 ns. We chose a cut-off value of 0.9 nm for van der Waals interactions. Constrains on bonds lengths and angles were applied with the LINCS algorithm. Periodic boundary conditions were used.

Coarsening of Biotin: from Gromos53a6 to MARTINI

The biotinyl termination of biotinylated BMHP1-derived SAPs was mapped and parametrized in accordance with the MARTINI approach. We adopted the coarsening approach proposed by Marrink et al. [2]. At first we mapped the chemical structure of biotinyl for a CG representation. As shown in **Table S1**, biotinyl termination was divided in five atom groups, or grains. This mapping was necessary to assign the appropriate CG particle type to each grain.[2] The second step was the selection of the appropriate bonded interactions: lastly we optimized the model by comparing it to the corresponding UA (Gromos53a6 ff) simulations. We compared the UA and CG simulations of BMHP1-derived SAPs 8-mers. After many rounds of model optimization, the value of the bond lengths and angles measure, was chosen as shown in **Table S2, S3, S4.**

Biotinyl atom groups	Grain number	Interaction site	Type	Atom
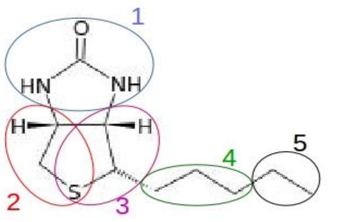	1	B61	P3	N2,C2,OB,N1
	2	B62	SC5	S,CF,CG
	3	B63	SC5	S,CE,CH
	4	B64	SC3	CO,CC,CB
	5	B65	Na	CA,C,O

Table S1. Biotinyl Coarse-grained mapping. The biotinyl group has been divided in five groups of atoms. In accordance with MARTINI mapping each group consists of 3 or 4 heavy atoms (C,N,O,S). In order to preserve cyclical geometry of ureido and thiophene ring, they were mapped respectively as a single interaction site (B61) and two different interaction sites (B62,B63). The valeryl chain was mapped with two interaction sites (B64, B65). The B65 interaction sites represent the amide group connecting valeryl chain to the first residue of BMHP1-derived SAPs.

Bond id	Bond Type	Bond length (Å)	Force costant (kJ mol $^{-1}$ nm^{-2})
b1 (1-2)	P3-SC5	0,32	constraint
b2 (2-3)	SC5-SC5	0,09	constraint
b3 (1-3)	P3-SC5	0,319	constraint
b4 (3-4)	SC5-SC3	0,4	2500
b5 (4-5)	SC3-Na	0,29	1250

Table S2. Calculated bond lengths (Å) in biotinyl groups. Sidechain bonds of the grains representing the biotin rings were introduced (b1,b2,b3) to preserve the cyclical geometry of biotinyl group. The lengths of these bonds are similar to those of Trp.[3]

Angle id	Angle Type	Angle (Degree)	Force costant (kJ mol^{-1})
a1 (1-2-3)	P3-SC5-SC5	81,91	constraint
a2 (2-1-3)	SC5-P3-SC5	16,16	constraint
a3 (1-3-4)	P3-SC5-SC3	105,84	25.0
a4 (2-3-4)	SC5-SC5-SC3	154,371	25.0
a5 (3-4-5)	SC5-C3-Na	102,734	25.0

Table S3. Calculated bead angles in biotin structure. In the CG model bead angles were introduced to mimic the atomic structure of biotin.

Dihedral id	Dihedral Type	Angle(Degree)
d1 (1-2-3-4)	P3-SC5-SC5-SC3	-0,61
d2 (3-1-2-4)	SC5-P3-SC5-SC3	-0,11
d3 (1-3-4-5)	P3-SC5-SC3-Na	-1,64
d4 (2-3-4-5)	SC5-SC5-SC3-Na	3,687

Table S4. Calculated dihedral angles in biotin structure. Dihedral angles d1, d2, d3 and d4 were chosen to preserve the relative orientation among ureido and thiophene rings, as well as their planar geometry.

Biotin partitioning coefficient

Following the MARTINI protocol for the parametrization of new molecules, we calculated the biotin octanol/water partition coefficient P_{OW}.[2][3] To obtain P_{OW}, the free energy of solvation of biotin was calculated in both aqueous and organic phase. The difference between the solvation free energy in the aqueous (ΔG^{W}) and organic phase (ΔG^{o}) is the partitioning free energy ($\Delta\Delta G^{OW}$) of biotin between water-saturated octanol solution and water:

$\Delta\Delta G^{oW}$ = -2.3 RTlogP_{ow}

R is the molar constant of Boltzmann, T is the temperature (set to 300 K), ΔG^{W} and ΔG^{o} are calculated as the free energy ΔF of the solute in vacuum (state A) and in the condensed phase (state B) using the thermodynamic integration (TI) approach.

$$\Delta F_{BA} = F_B - F_A = \int_{\lambda_A}^{\lambda_B} d\lambda \left\langle \frac{\delta U_{uv}(\lambda)}{\delta\lambda} \right\rangle_{\lambda}$$

$U_{uv}(\lambda)$ denotes the potential energy function describing the total solute-solvent interaction: the average $<\frac{\delta U_{uv}(\lambda)}{\delta\lambda}>$ is calculated over the MD trajectory. U_{uv} is a function of λ (the coupling parameter) and varies linearly from 0 to 1. Calculations were performed at 20 intermediates λ values.

Biotin atom types used for thermodynamic integration are reported in Table S1: however, atom type 5 had to be changed from Na to P3 (protonated carboxyl group). Indeed, in order to reproduce experimental conditions for partition coefficient calculation,[4] biotin was assumed to be fully protonated (pKa = 4,4 , pH≈4 in distilled water). The thermodynamic integration simulation setup and the calculated solvation free energies (ΔG^{W},ΔG^{o}) are reported in table S5. A s shown in Table S5 biotin CG model is satisfactorily parametrized as it reproduces biotin thermodynamic properties.

Biotin	Water	Octanol	Time(ns)	T (K)	ΔG_{SOLV} (kJ/mol*K)	
1	399	0	20 x 6	300	82,51	**ΔG^{W}**
1	0	181	20 x 6	300	84,87	**ΔG^{O}**
					2,36	**ΔG^{OW}**
		**$logP_{OW}(CG)$**<br>-0,411			**$logP_{OW}(exp)$**<br>-0,462	

Table S5. Summary of the simulation performed for TI and related results. The difference between the calculated (**$logP_{OW}(CG)$**) and the experimental (**$logP_{OW}(exp)$**) partition coefficients suggests a good reproduction of the thermodynamic properties of biotin with the MARTINI coarsened model we developed.

**Shift Value (k)**	**Parallel arrangement (P)**	**Antiparallel arrangement with positive shift (A+)**	**Antiparallel arrangement with negative shift (A-)**
0	NtGGGX_1FX_2STKT<br>NtGGGX_1FX_2STKT	NtGGGX_1FX_2STKT<br>TKTSX_2FX_1GGGN_t	NtGGGX_1FX_2STKT<br>TKTSX_2FX_1GGGN_t
1	NtGGGX_1FX_2STKT<br>NtGGGX_1FX_2STKT	NtGGGX_1FX_2STKT<br>TKTSX_2FX_1GGGN_t	NtGGGX_1FX_2STKT<br>TKTSX_2FX_1GGGN_t
2	NtGGGX_1FX_2STKT<br>NtGGGX_1FX_2STKT	NtGGGX_1FX_2STKT<br>TKTSX_2FX_1GGGN_t	NtGGGX_1FX_2STKT<br>TKTSX_2FX_1GGGN_t
3	NtGGGX_1FX_2STKT<br>NtGGGX_1FX_2STKT	NtGGGX_1FX_2STKT<br>TKTSX_2FX_1GGGN_t	NtGGGX_1FX_2STKT<br>TKTSX_2FX_1GGGN_t
4	NtGGGX_1FX_2STKT<br>NtGGGX_1FX_2STKT	NtGGGX_1FX_2STKT<br>TKTSX_2FX_1GGGN_t	NtGGGX_1FX_2STKT<br>TKTSX_2FX_1GGGN_t
5	NtGGGX_1FX_2STKT<br>NtGGGX_1FX_2STKT	NtGGGX_1FX_2STKT<br>TKTSX_2FX_1GGGN_t	NtGGGX_1FX_2STKT<br>TKTSX_2FX_1GGGN_t
6	NtGGGX_1FX_2STKT<br>NtGGGX_1FX_2STKT	NtGGGX_1FX_2STKT<br>TKTSX_2FX_1GGGN_t	NtGGGX_1FX_2STKT<br>TKTSX_2FX_1GGGN_t
7	NtGGGX_1FX_2STKT<br>NtGGGX_1FX_2STKT	NtGGGX_1FX_2STKT<br>TKTSX_2FX_1GGGN_t	NtGGGX_1FX_2STKT<br>TKTSX_2FX_1GGGN_t
8	NtGGGX_1FX_2STKT<br>NtGGGX_1FX_2STKT	NtGGGX_1FX_2STKT<br>TKTSX_2FX_1GGGN_t	NtGGGX_1FX_2STKT<br>TKTSX_2FX_1GGGN_t
9	NtGGGX_1FX_2STKT<br>NtGGGX_1FX_2STKT	NtGGGX_1FX_2STKT<br>TKTSX_2FX_1GGGN_t	NtGGGX_1FX_2STKT<br>TKTSX_2FX_1GGGN_t
10	NtGGGX_1FX_2STKT<br>NtGGGX_1FX_2STKT	NtGGGX_1FX_2STKT<br>TKTSX_2FX_1GGGN_t	NtGGGX_1FX_2STKT<br>TKTSX_2FX_1GGGN_t

Table S6. Shift values of BMHP1-derived SAPs. Possible β-strands alignments within β-sheets of BMHP1-derived SAPs. N_t denotes N-terminal acetylation (Ac) or functionalization with Biotin(Btn) or the amino acid residue tryptophan (Ac-W). X_1 denotes phenylalanine (P) or alanine (A). X_2 stands for serine (S) or alanine (A). Only one type of parallel arrangement is represented as there are no differences among parallel arrangements with positive and negative shifts.

Shift Value (k)	Parallel arrangement	Antiparallel arrangement
**0**	LDLKLDLKLDLK<br>LDLKLDLKLDLK	LDLKLDLKLDLK<br>KLDLKLDLKLDL
**1**	LDLKLDLKLDLK<br>LDLKLDLKLDLK	LDLKLDLKLDLK<br>KLDLKLDLKLDL
**2**	LDLKLDLKLDLK<br>LDLKLDLKLDLK	LDLKLDLKLDLK<br>KLDLKLDLKLDL
**3**	LDLKLDLKLDLK<br>LDLKLDLKLDLK	LDLKLDLKLDLK<br>KLDLKLDLKLDL
**4**	LDLKLDLKLDLK<br>LDLKLDLKLDLK	LDLKLDLKLDLK<br>KLDLKLDLKLDL
**5**	LDLKLDLKLDLK<br>LDLKLDLKLDLK	LDLKLDLKLDLK<br>KLDLKLDLKLDL
**6**	LDLKLDLKLDLK<br>LDLKLDLKLDLK	LDLKLDLKLDLK<br>KLDLKLDLKLDL
**7**	LDLKLDLKLDLK<br>LDLKLDLKLDLK	LDLKLDLKLDLK<br>KLDLKLDLKLDL
**8**	LDLKLDLKLDLK<br>LDLKLDLKLDLK	LDLKLDLKLDLK<br>KLDLKLDLKLDL
**9**	LDLKLDLKLDLK<br>LDLKLDLKLDLK	LDLKLDLKLDLK<br>KLDLKLDLKLDL
**10**	LDLKLDLKLDLK<br>LDLKLDLKLDLK	LDLKLDLKLDLK<br>KLDLKLDLKLDL
**11**	LDLKLDLKLDLK<br>LDLKLDLKLDLK	LDLKLDLKLDLK<br>KLDLKLDLKLDL

Table S7. Shift values of LDLK-derived SAPs. The mutual alignment among $(LDLK)_3$ SAP or CAPs can be summarized in parallel or antiparallel alignments without distinction between positive and negative shifts.

Cluster	Cluster population	SS parameters
1	4	CCSSCCCCCC
2	2	CCCCCCCCCC
3	1	CCSCCCTTCC
4	1	CCSSCCSSCC

Table S8. SAM Secondary structure parameters from conformational sampling of B24. Secondary structure parameters were identified through the analysis of monomer UA-MD simulations. The secondary structure of the corresponding monomer has been monitored by means of DSSP algorithm of Kabsch and Sander.[1]

Cluster	Cluster population	SS parameters
1	3	CETTECCCCCC
2	2	CEETTTEECCC
3	1	CCSSCCCSCCC
4	1	CCCSCCCCCCC
5	1	CCSSCCSSSCC

Table S9. SAM Secondary structure parameters from conformational sampling of 30. The secondary structure parameters of 30 were identified following the same workflow adopted for B24. The 30 monomer starting conformations in octameric systems were more polymorphic compared to B24.

Cluster	Cluster population	SS parameters
1	2	CCSSSCCCCCC
2	2	CCCSSCCCCCC
3	1	CCSSSSCCCCC
4	1	CCSSSCSSSCC
5	1	CCSSCCCSSCC
6	1	CCCCCCSSSCC

Table S10. SAM Secondary structure parameters from conformational sampling of 31. As reported in our previous work, secondary structure parameters were monitored, in monomer UA-MD simulations, by means of the DSSP algorithm.[1] If compared to octameric systems of B24 and 30, the monomer starting conformations of 31 were more polymorphic.

Supplementary Figures

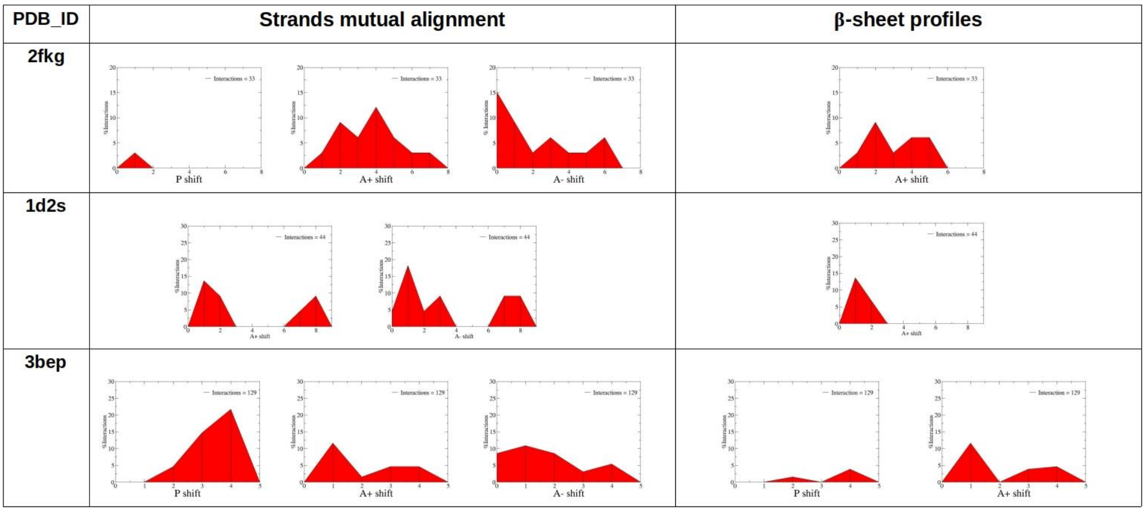

Figure S1. Shift profiles of the proteins shown in Fig.1. Different PDB structures, subsequently CG-mapped, were analysed through Morphoscanner. Shift profiles indicated anti-parallel alignments of strands for 2fkg. This folding was reflected in β-sheet structures: indeed, β-strands featured mainly A+ anti-parallel alignments. In 1d2s mainly anti-parallel out-of-register strands and β-sheets were seen. The organization of 3bep was more complex: indeed, most strands followed a parallel alignment, while β-strands were preferentially anti-parallel aligned out-of-register by one-residue.

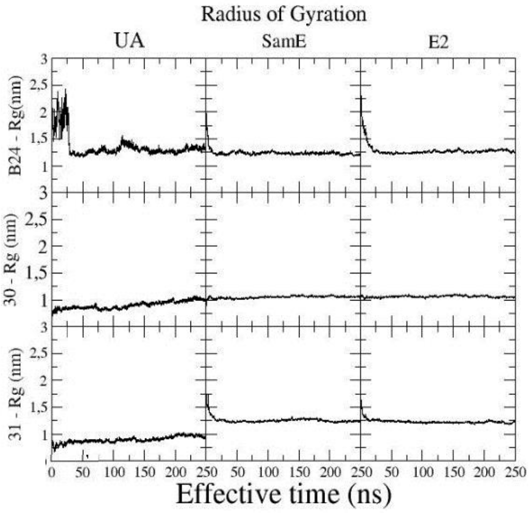

Figure S2. Radius of gyration of octameric systems in UA-MD and CG-MD simulations. As reported in our previous work, [1] the nucleation in UA-MD simulations occurred within 50 ns. The CG simulations of octameric systems with fully extended secondary structure parameters denoted a similar tendency in the nucleation feature of SAP systems. Indeed, the aggregate sizes of octameric systems were similar regardless of the chosen simulation model.

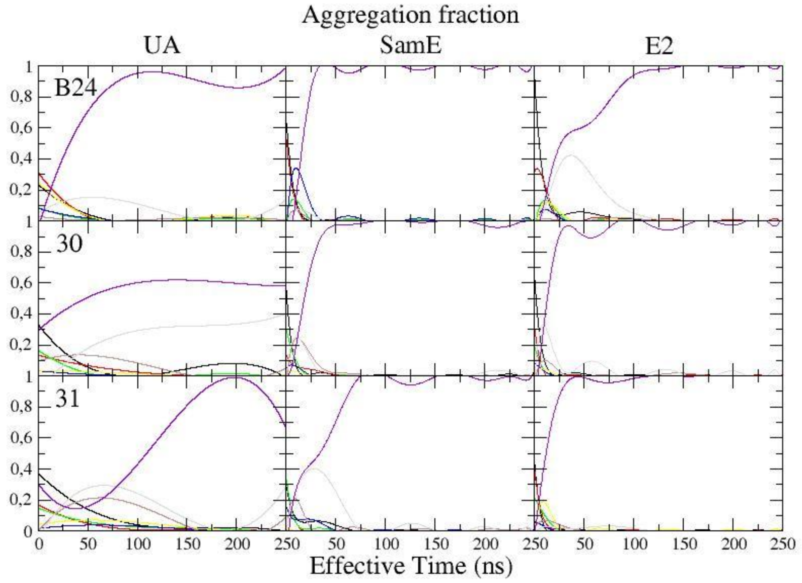

Figure S3. Aggregation fraction of the multi-mers composing each system: 8-mer in purple, 7-mer in silver, 6-mer in grey, 5-mer in blue, 4-mer in yellow, 3-mer in green, 2-mer in red, 1-mer in black. UA-MD and CG-MD simulations refer to octameric systems containing NaCl 0.015 M. The starting conformations of monomers were Sam (sampled from the UA-MD simulation of monomer) or E (fully extended). In CG-MD simulations with E secondary structure parameters oligomerization mechanism proceeded through similar intermediate steps unrelated to the initial monomeric conformation. These tendencies were similar to those observed in UA-MD simulations.

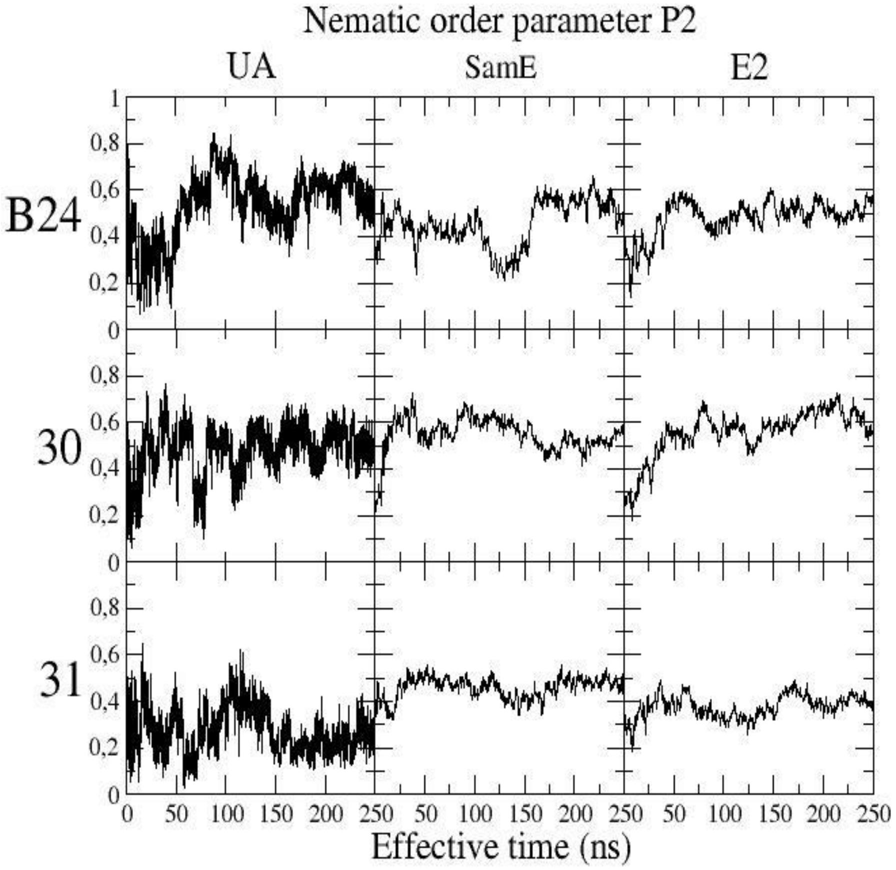

Figure S4. Nematic order parameter P2 of octamers of B24,30,31. In CG-MD simulations (SS parameters=E) the nematic order parameter P2 reached similar values observed in UA-MD simulations regardless of the initial monomeric conformations.

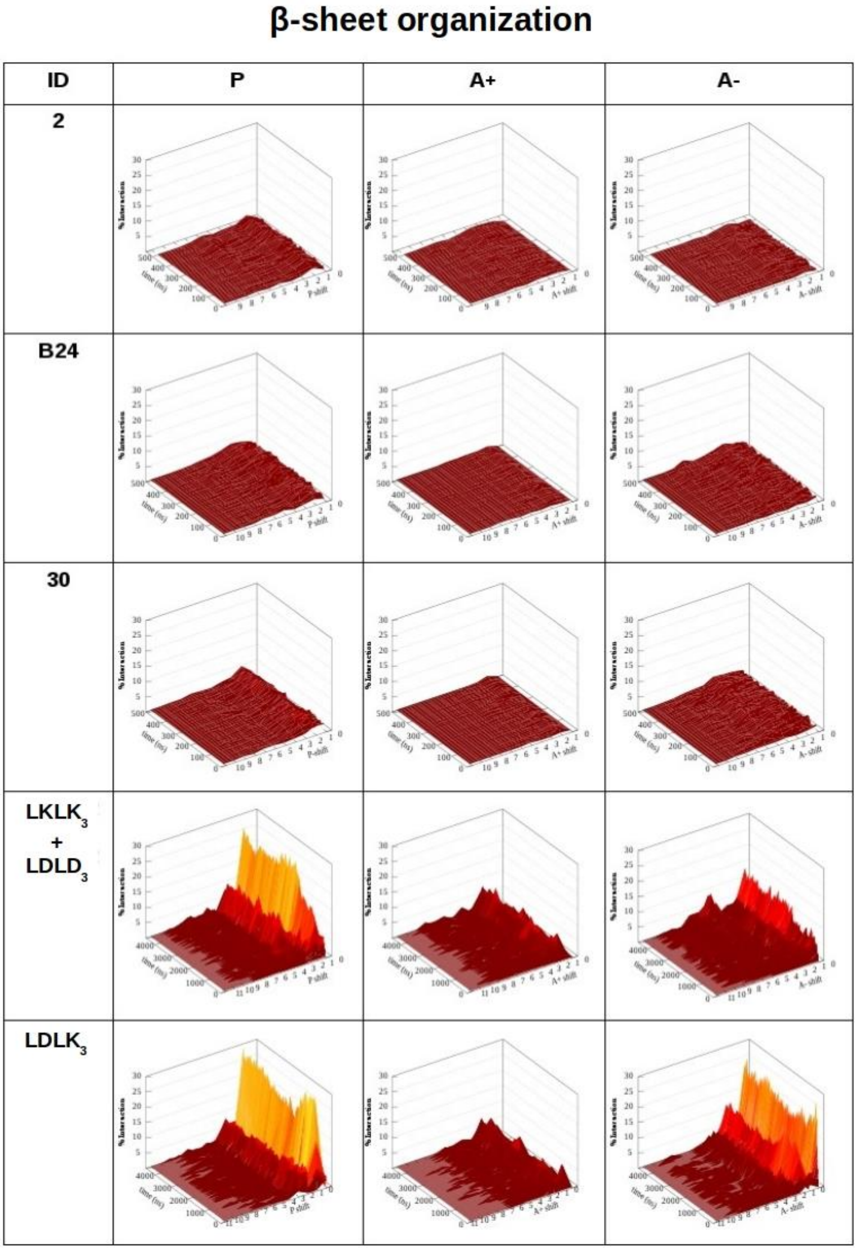

Figure S5. Shift profiles within β-sheet alignments. CAPs and $(LDLK)_3$ assembled in stable β-sheet structures. Instead, BMHP1-derived SAPs did not show relevant formation of β-sheets. SAP 2, B24 and 30 were simulated with fully extended secondary structure parameters (see Table 1).

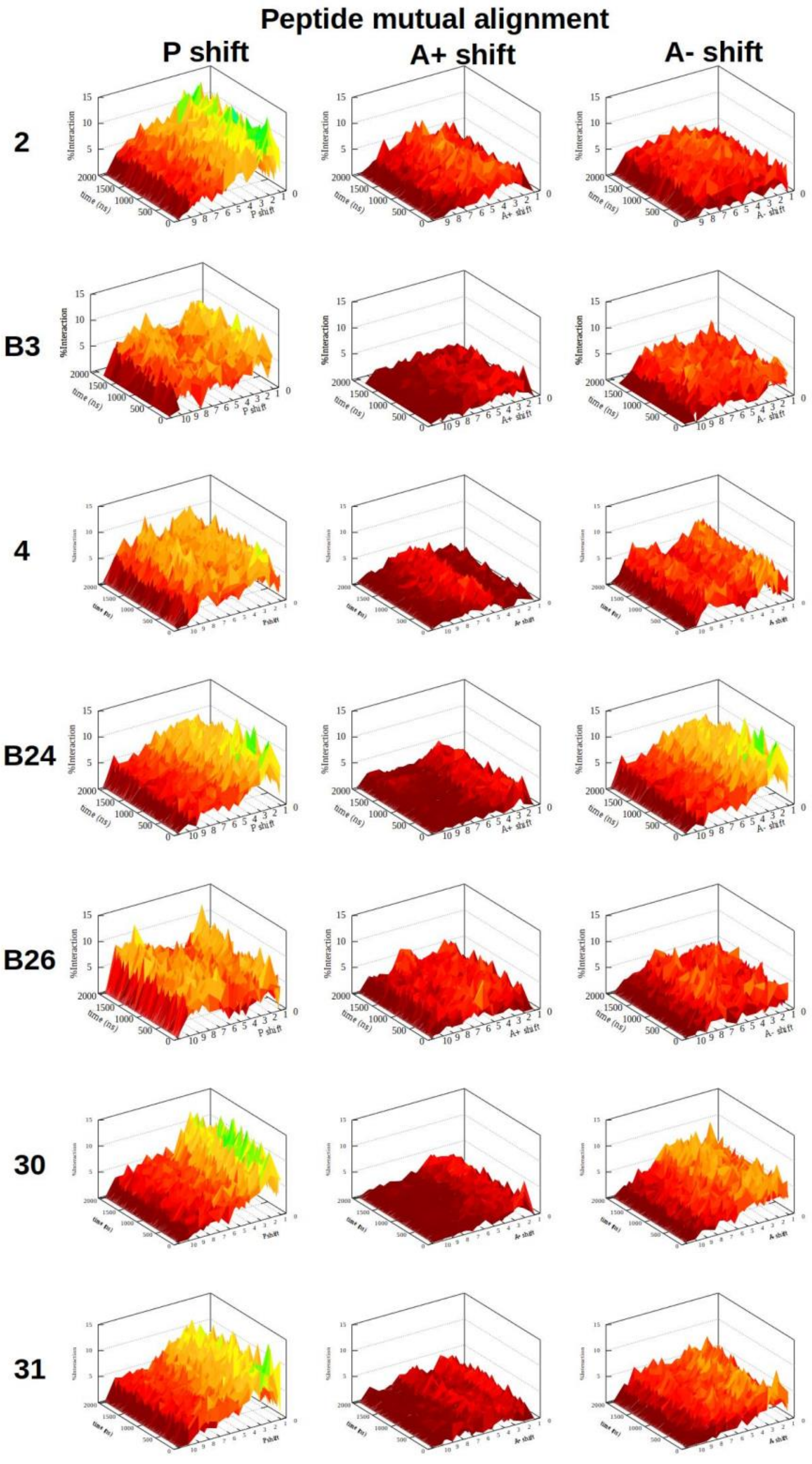

Figure S6. BMHP1-derived SAPs mutual alignment. Analysis of CG-MD simulations with full extended MARTINI SS parameters. BMHP1-derived SAPs were preferentially aligned at one-residue shift in parallel arrangements. Notably, shift profiles of SAPs with Pro privileged parallel shift out-of-register by one-residue (2,B3,4,B26), while mutation with Ala coaxed the onset of A- shift (B24,30,31).

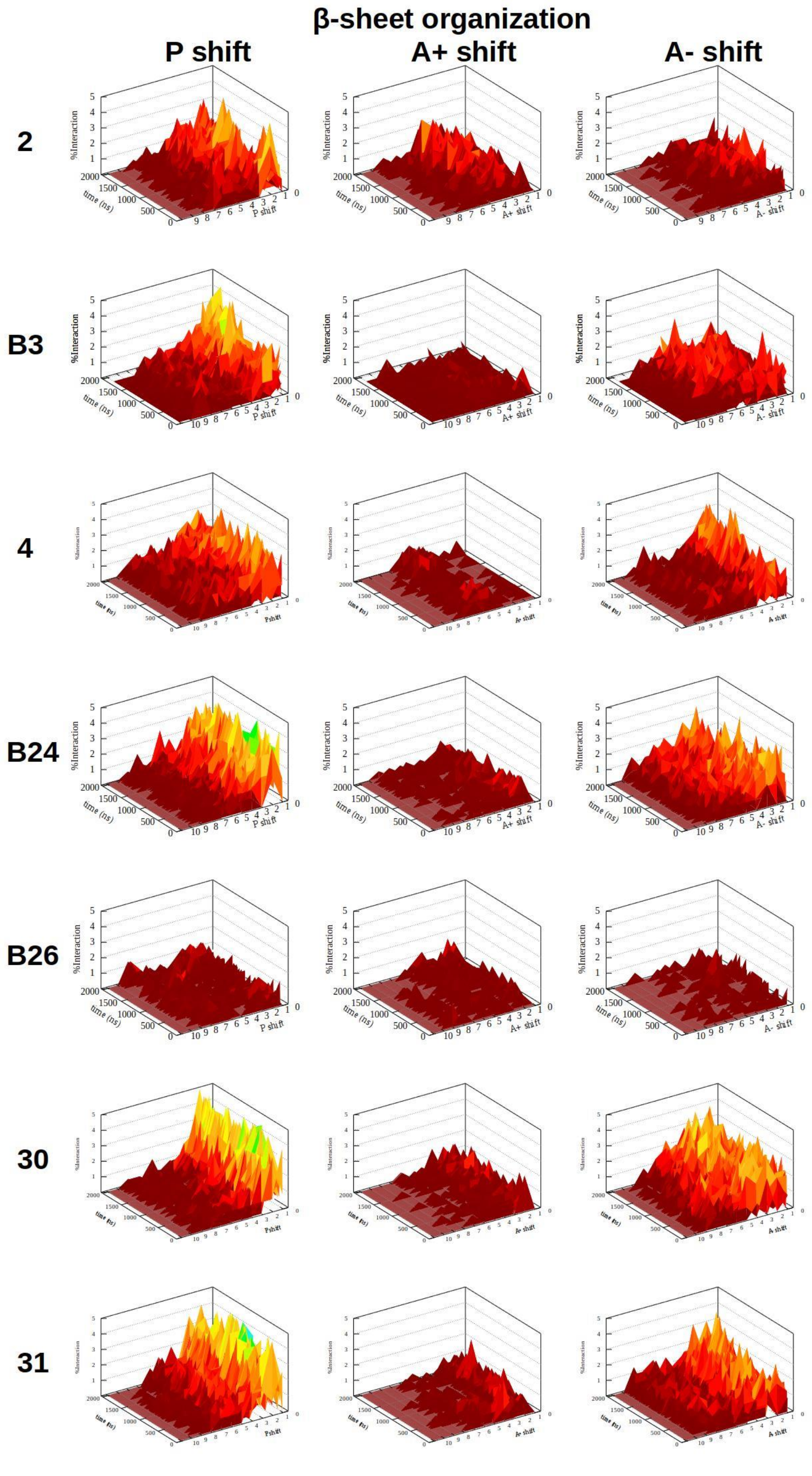

Figure S7. β-sheet organization of BMHP1-derived SAPs. BMHP1-derived SAPs preferentially aligned at one-residue shift in parallel arrangements within β-sheets. B26 showed the poorest propensity to β-sheet organization.

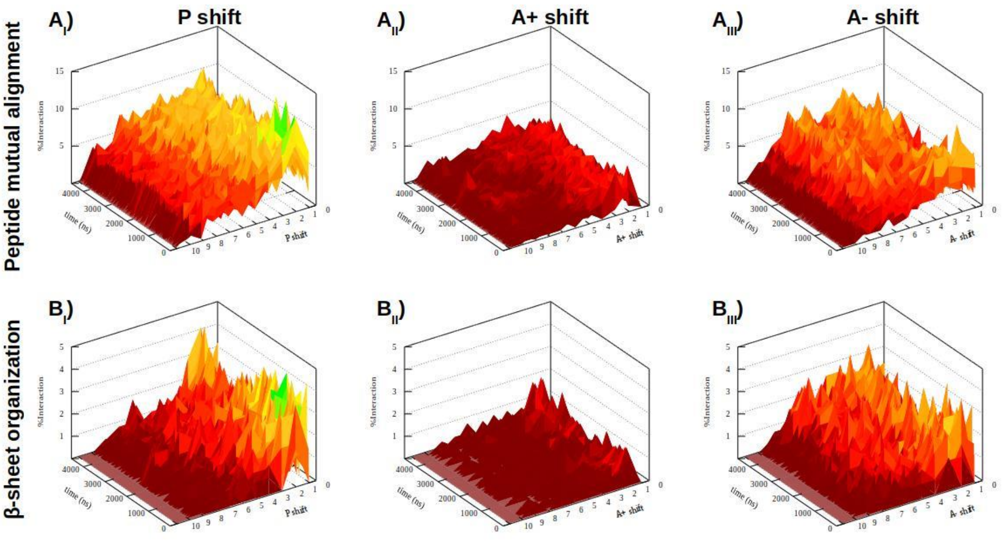

Figure S8. Shift profiles of B24 (SS parameters=E). The analysis of peptide mutual alignment and β-sheet organization were extended till 4500 ns for B24. Shift profiles showed that B24 preferentially aligned in parallel out-of-register but were not centered around any particular value, therefore B24 oligomers were characterized by a heterogeneous alignment. Shift profiles of β-sheet organization showed that β-strands were mutually aligned in parallel out-of-register.

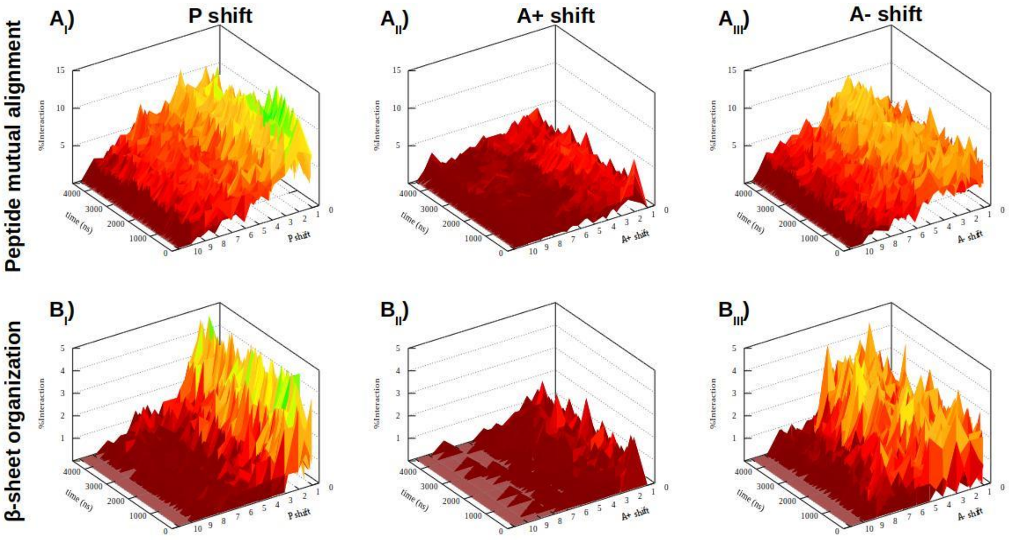

Figure S9. Shift profiles of SAP 30 (SS parameters=E). Shift profiles of CG-MD simulations of peptide 30 for up to 4500 ns. In $A_{I\text{-}III}$ peptides were preferentially aligned in parallel out-of-register by one-residue. Same situation could be appreciated in $B_{I\text{-}III}$ for β-sheet organization. 30 showed a lower tendency to β-structuring if compared to B24. This feature is likely given by the presence of Trp at the N-terminal.

References.

[1] G. Saracino, F. Gelain, *Modeling and analysis of early aggregation events of BMHP1-derived self-assembling peptides,* Journal of Biomolecular Structure and Dynamics, 2013, DOI:10.1080/07391102.2013.790848

[2] S. J. Marrink, H. J. Risselada, S. Yefimov, D. P. Tieleman, A. H. de Vries, *The MARTINI Force Field: Coarse Grained Model for Biomolecular Simulations*, J. Phys. Chem. B, 2007, 111, 7812-7824

[3] L. Monticelli, S. K. Kandasamy, X. Periole, R. G. Lanson, D. P. Tieleman, S. J. Marrink, *The MARTINI Coarse-grained Force Field: Extension to Proteins*, J. Chem. Theory and Comput., 2008, 4, 819-834

[4] F. Della Valle, S. Lorenzi, G. Calderini, Water soluble derivatives of biotin and related therapeutical compositions, US Patent 5550249
